# Floating proximal interphalangeal joint (PIPJ) injury of the little finger in a child: A case report

**DOI:** 10.1016/j.tcr.2021.100458

**Published:** 2021-03-17

**Authors:** Sonu Mehta, Jagan John Jacob John, Thayur R. Madhusudhan

**Affiliations:** Trauma & Orthopaedics, Glan Clwyd Hospital, Rhyl (Betsi Cadwaladr University Health Board), United Kingdom of Great Britain and Northern Ireland

**Keywords:** Floating proximal interphalangeal joint, Sub-condylar phalanx fracture, Fracture neck phalanx

## Abstract

We present a floating PIPJ injury of the non-dominant hand little finger in a skeletally immature boy following a hyperextension injury while playing football. The injury was managed non-operatively with a successful outcome. This injury pattern can happen with trivial trauma in a child and could be easily missed. It is important to be aware of this pattern of injury and good functional outcomes are possible with non-operative treatment.

## Introduction

Hand injuries in children usually present in a varied pattern with most injuries occurring around the phalanges. These fractures are epiphyseal injuries and normally confined to individual digits and bones.

We report a case of fracture of neck of proximal phalanx along with fracture of base of middle phalanx of the little finger in a 15 year old child following a football injury. Though similar injuries have been reported around metacarpophalangeal joints in adults [1,2], we were unable to find similar injury pattern in children in published English literature. The report here is presented for its occurrence in children and the importance of the clinician in being aware of this injury. The injury was managed non- operatively with good outcome.

## Case report

**Fig. 1:**
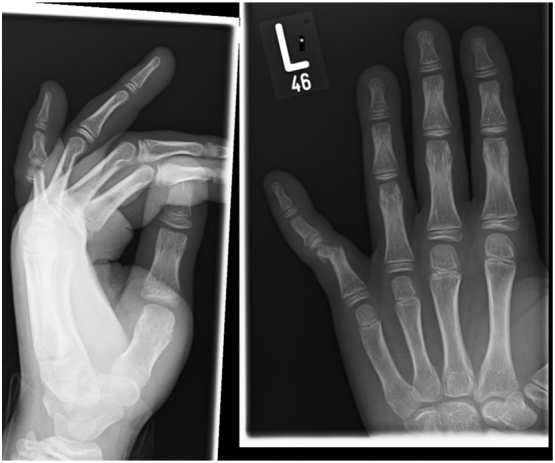
At first assessment.

A 15 year old boy presented to the emergency department with a history of hyperextension injury to the left little finger while playing football. On assessment, it was noted that the little finger was swollen, bruised on the volar aspect and deformed. The injury was closed and there were no distal neurovascular concerns. There were no other associated skeletal injuries. Further radiographs of the hand confirmed a transverse fracture through the neck of the little finger proximal phalanx with ulnar displacement of the proximal phalangeal head and also an undisplaced fracture of the basal metaphysis of the middle phalanx (Fig. 1).

**Fig. 2:**
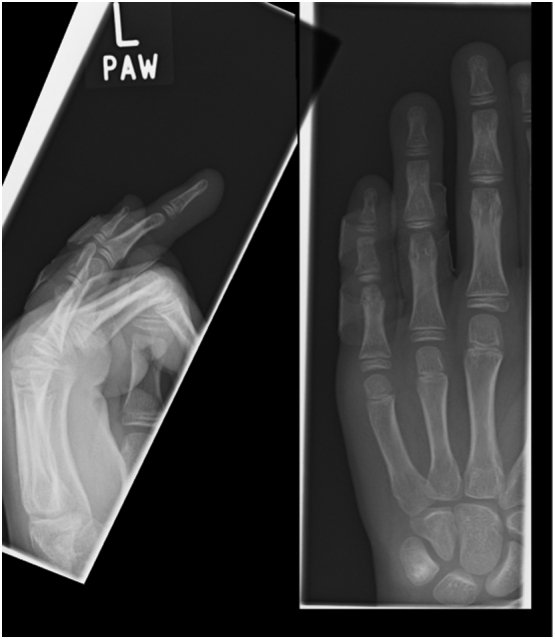
2 weekly radiograph.

Under adequate analgesia and digital anaesthetic block the fracture was manipulated to a satisfactory alignment and maintained with neighbour strapping and a dorsal extension block splinting at 30 degrees flexion. Post reduction radiographs revealed an improvement in the position of the head of the proximal phalanx with unchanged appearance of the middle phalangeal fracture. The PIPJ alignment was satisfactory. Clinically the injury was stable and there was no change in the neurovascular status. The patient was further followed up in the fracture clinic and reduction was assessed both clinically and radiologically with serial radiographs for 2 weeks since the injury. At 4 weeks, dorsal extension block splinting was discontinued and clinical healing was confirmed. The patient was discharged to physiotherapy and further assessed at 8 and 12 weeks. By 12 weeks, the injury had fully healed with mild ulnar deviation at the proximal phalanx but with complete restoration of function (Fig. 2).

## Discussion

**Fig. 3:**
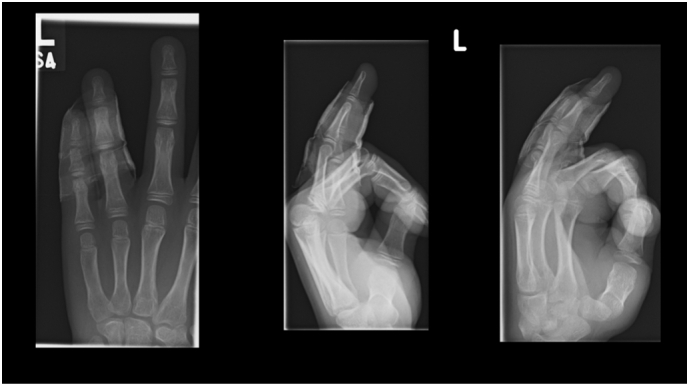
4 weekly radiograph.

Phalangeal fractures are the most common reported hand fractures in the paediatric age group especially between age group of 10–14 years after they start playing contact sports [3]. The common mechanism of injury in these fractures is believed to be due to axial loading combined with torsional or angular force. In the present case the digit was injured while trying to stop the ball with an open hand suggesting a hyperextension injury pattern combined with a probable axial loading [4]. Boys do account for a higher incidence of these injuries, probably owing to preferential involvement in contact sports but the footfall of Paediatric phalanx fractures in speciality hand clinics in UK is only 264/100,000 children implying that most of them do get treated definitively in the emergency department [5,6]. It is therefore important for the emergency room clinicians to recognise this injury pattern and refer them to appropriate specialty clinics for better outcomes (Fig. 3).

**Fig. 4:**
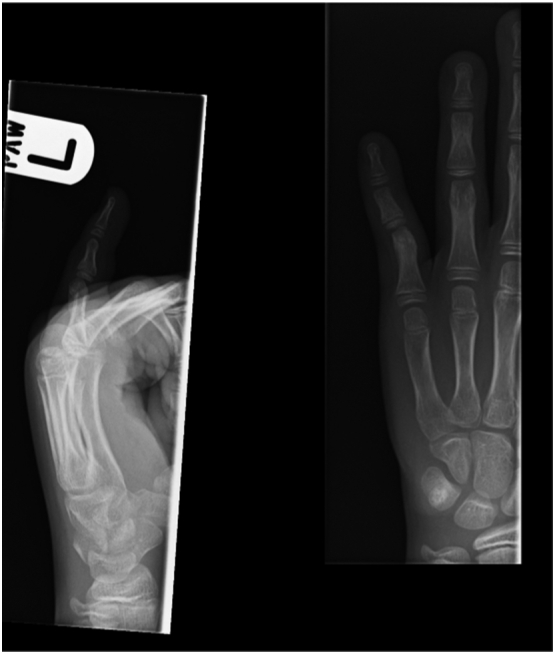
3 monthly radiograph.

Extension of the PIPJ places a strain on the volar plate, and in hyper extension the volar plate may fail either by rupture or avulsion of the volar lip at the middle phalanx [7], in adults. In the paediatric patient, unmineralized physis is biomechanically weaker than the surrounding ligamentous structures and mature bone, which makes fractures about the physis more likely compared with ligamentous injuries or diaphyseal fractures [3]. The floating nature of the injury pattern suggests severity of the impact on the finger. The physes are located on the proximal aspect in the phalanges unlike the metacarpals where they are in the distal segment (Fig. 4).

Due to the differential growth, the injuries around the physes may have exaggerated displacement at the outset but have an excellent remodelling potential in the plane of motion, i.e. flexion/extension [8]. The head fragment due to attachment of the collateral ligaments usually goes into extension which may cause the volar spike to create a block in flexion of the IP joint [4]. Extension block splinting allows relaxation of the collateral ligaments which aids in correcting the deformity and an intact volar plate provides additional stability to the PIPJ. The middle phalanx fracture position did not change following manipulation suggesting an inherent stability of the soft tissues around the PIPJ and hence we continued to manage non-operatively.

**Fig. 5:**
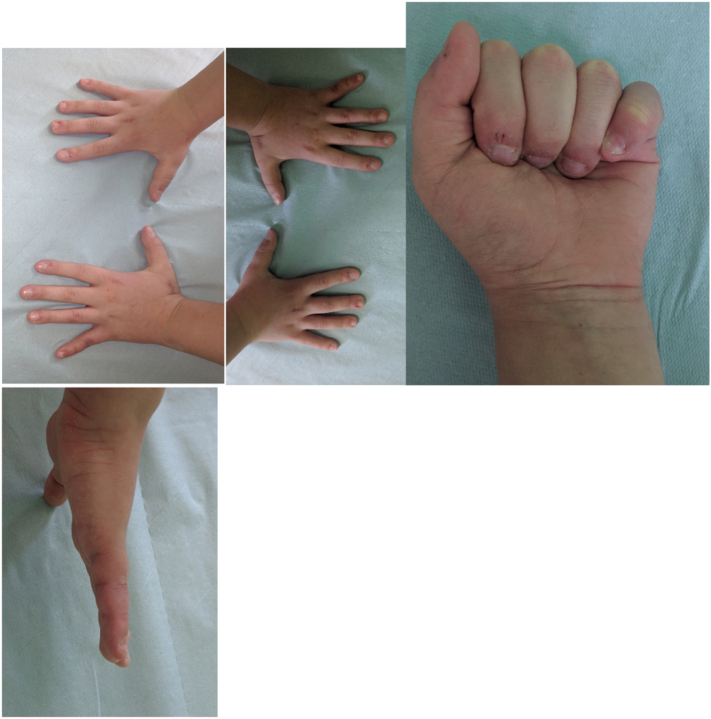
Clinical pictures at 3 months.

Management of isolated proximal phalangeal neck fractures is controversial with few studies suggesting Closed reduction and pinning [9] with most requiring formal hand physiotherapy. Similar outcomes between non-operative and operative methods of fixation [10] have been suggested, when dorsal extension block splinting has been elected as a definitive treatment for these injuries. Closed treatment of sub condylar fractures of the proximal phalanx is difficult as these are often unstable. They have a tendency to redisplace into extension unless securely fixed via percutaneous pins or open reduction (Fig. 5).

A paediatric patient presents several unique challenges – Difficult communication, well oriented radiographs may be difficult to obtain, overlapping digits may obscure radiological assessments, smaller size of the bone and digit may preclude surgical fixation etc. A surgical option in our patient would have necessitated violation of the PIPJ with added risks of chondral and or physeal damage. We noticed that the distal fragment of the proximal phalanx did reveal a tendency to slip back into extension during serial radiographic examination but this did not seem to affect the range of movements in the fractured digit eventually. It is therefore important that these injuries are closely monitored until the healing is complete. Even though at final discharge, functional outcomes were good in terms of stability and range of movements, we cannot comment on the long term outcomes at this point. Parent counselling regarding the injury is therefore important in these rare and complex injury patterns in the paediatric patient.

## Conclusion

Floating proximal interphalangeal joint hand injury is a rare complex pattern which can occur with trivial injury in children. Closed treatment is successful achieving good outcomes and requires closed monitoring in specialty clinics.

## Declaration of competing interest

The authors do not report any conflict of interest.
